# Development of Pore Pressure in Cementitious Materials under Low Thermal Effects: Evidence from Optimization of Pore Structure by Incorporation of Fly Ash

**DOI:** 10.3390/ma16124214

**Published:** 2023-06-06

**Authors:** Wei Jiang, Dandan Zhang, Xinyue Zheng, Wenqian Li

**Affiliations:** 1 Key Laboratory of Advanced Civil Engineering Materials of Ministry of Education, School of Materials Science and Engineering, Tongji University, Shanghai 201804, China; 2 School of Materials Science and Engineering, Tongji University, Shanghai 201804, China

**Keywords:** durability, fly ash, low thermal loading, pore pressure, numerical simulation

## Abstract

Studies on durability of cementitious materials have focused on harsh environments, but less attention has been paid to low thermal loading situations. In this paper, with the aim of exploring the evolution of internal pore pressure and microcrack extension of cementitious under low thermal environment, cement paste specimens with thermal environment slightly below 100 °C and three water–binder ratios (0.4, 0.45 and 0.5) and four fly ash admixtures (0, 10%, 20% and 30%) were designed. Firstly, the internal pore pressure of the cement paste was tested; secondly, the average effective pore pressure of the cement paste was calculated; and finally, the phase field method was used to explore the expansion of microcracks inside the cement paste when the temperature gradually increased. It was found that the internal pore pressure of the paste showed a decreasing trend as the water–binder ratio and fly ash admixture increased, and the numerical simulation found that the sprouting and development of cracks were delayed when 10% fly ash was added to the cement paste, which was consistent with the experimental results. This work provides a basis for the durability development of concrete under low thermal environment.

## 1. Introduction

Concrete is the most widely used construction material in the world today; however, during its service, carbonation, sulfate attack, steel corrosion, freeze–thaw cycles, and alkali aggregate reactions occur, all of which can lead to deterioration in the durability performance of concrete materials [1,2,3]. The nature of durability deterioration of concrete materials is due to the internal physicochemical reactions that lead to changes in the internal relative humidity and pore structure, and the pore pressure in the pore structure changes with changes in the internal humidity and pore radius of the pores. As the pore pressure inside the concrete structure increases, its internal organization gradually loosens, pores expand, and cracks occur, causing the expansion of microcracks, thus decreasing the durability of the material [4,5,6,7]. According to the theory of pore elastodynamics, pore pressure acting on the material inevitably causes strain in the material, and the macroscopic strain of the material is the sum of the strain caused by temperature strain and pore stress [8,9,10]. Therefore, an in-depth understanding of the pore pressure mechanism of concrete materials not only has research significance for its scientific problem of microcrack expansion but is also an important basis for preventing or improving the durability of concrete materials in engineering.

The current research on the pore pressure of concrete materials is mainly focused on the field of high-temperature bursting, such as the studies that found that when warming, the initial small cracks inside the concrete gradually develop into penetration cracks [11]; with the increase in temperature, the pore vapor pressure gradually rises, and its peak can reach 0.2~1 MPa [12]; the peak pore vapor pressure and the concrete strength grade, moisture content size, warming rate, and compactness are closely related [13]; and when the crack penetration provides a channel for the release of its pore vapor pressure, the vapor pressure decreases [14]. It seems that the research related to the pore pressure of concrete is mainly focused on the investigation of the mechanism at high temperatures, and the research on the pore pressure of concrete at temperatures below 100 °C is still relatively lacking; however, the changes in the pore pressure inside concrete at room temperature can also cause microdamage to the material, thus affecting its durability. Therefore, it is necessary to study the pore pressure of concrete at room temperature.

In this paper, firstly, by analyzing the test apparatus regarding the internal pore pressure test of concrete materials at high temperature, cement paste test blocks with 0~30% fly ash admixture were designed and the cement-paste pore structure optimized with fly ash [15,16]. This paper sets up a thermal environment from room temperature to slightly below 100 °C to quantify the internal pore pressure values of cement paste. Secondly, the pore structure parameters, such as porosity, average pore size, and pore surface area, as well as different pore size distribution functions of the cement paste test blocks were tested by mercury-pressure experiments, and the pore characteristic size R of the cement paste was discovered by establishing the size distribution function of the pores, and the average effective pore pressure P under a certain temperature T and relative humidity RH conditions was calculated theoretically [17] to explore and analyze the variation law of the internal pore pressure from the microscopic perspective on the durability of the net paste. Finally, the phase field method was used to simulate the crack development, and the pore pressure corresponding to the crack emergence and penetration was solved by randomly placing holes with a corresponding volume share according to the pore size probability distribution, and the pore-pressure-variation law was analyzed by comparing the experimental data.

## 2. Materials and Experimental Procedures

### 2.1. Materials and Matching Ratio

#### 2.1.1. Materials

In this study, PII52.5 silicate cement with stable properties and fly ash were used to form the slurry, and its chemical composition is shown in Table 1.

#### 2.1.2. Matching Ratio

Three water–binder ratios, 0.4, 0.45 and 0.5, and four fly ash blends, 0%, 10%, 20% and 30% mass fraction, were designed for this thesis test. The pore structure of the net cement paste is changed by changing the water–binder ratio and fly ash admixture. The reasons for this are as follows: (1) Different water–binder ratios provide different growth spaces for hydration products. When the water–binder ratio decreases, it can promote the crystallization, nucleation and crystal growth of hydration products, and promote the degree of hydration reaction in the early stage of cement paste, which eventually leads to the change in pore structure of cement paste system; (2) the active ingredients SiO_2_ and Al_2_O_3_ in fly ash react with Ca(OH)_2_ generated by the hydration of cement to produce a large amount of hydrated silicate gel, and some of its external hydration products will enter the particle voids, filling the voids and destroying the selective orientation arrangement of Ca(OH)_2_ in the interfacial area, and almost all of these above reactions are carried out in the pores of cement paste, so it can obviously change the porosity and pore structure of cementitious materials. The test pieces were classified and named and the fitting ratios are shown in Table 2. The size of the cement paste test pieces prepared for the test was 7 cm × 7 cm × 7 cm.

### 2.2. Design of Pore Pressure Measurement Test

#### 2.2.1. The Preparation Process of Cement Paste Test Block

**Figure 1:**
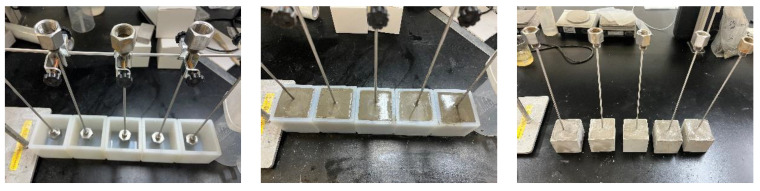
The process of making cement paste test block.

In this thesis, a total of 12 cement paste specimens for pore pressure testing were prepared using the raw materials and proportions of cement paste, and the preparation process of cement paste specimens is shown in Figure 1 [18]. To effectively test the variation in pore pressure inside the cement paste specimens, an air-conducting capillary tube with a metal screen was pre-buried at a distance of 35 cm from the heated surface of the specimens. The obtained cement paste specimens were cured after 24 h and immediately transferred to a maintenance room at 20 ± 2 °C and 95% relative humidity for 7 days. The test blocks were then dried with paper towels and placed in a ventilated and dry place for 1 h to test the internal pore pressure of the cement paste test blocks at high temperatures.

#### 2.2.2. Test Device and Method

Theoretically, the denser the cementitious material is and the lower the permeability is, the easier it is to generate pressure gradients and vapor pressures when it is heated. To effectively test the variation in internal pore pressure of 12 groups of cement paste test blocks with different ratios under the action of high temperature, this experiment refers to the literature [19,20] and designs a device specifically for independently testing the internal pore pressure of concrete under high temperature. The device consists of four main parts: a diffusion silicon high-temperature pressure transmitter, paperless recorder, digital display type electric furnace, and stainless steel air-conducting capillary tube with a metal filter. The principle is to pre-bury a stainless steel gas-conducting capillary tube inside the cement paste test block in advance and fill the capillary tube with silicone oil with relatively low thermal expansion before the test. When the cement paste test block is heated, its internal pore pressure will generate a certain pressure on the silicone oil inside the tube, which is then converted to the paperless recorder data collection device through a diffusion silicon high-temperature pressure transmitter.

**Figure 2:**
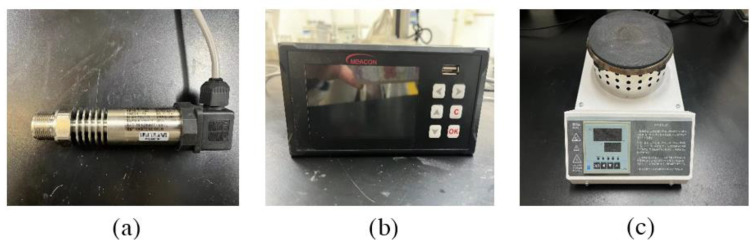
Testing equipment: (**a**)pressure transmitter, (**b**) paperless recorder and (**c**) electric heater.

**Figure 3:**
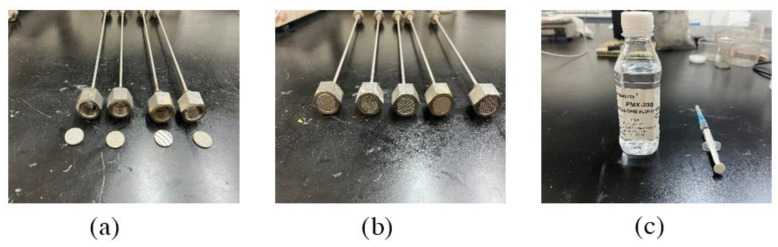
Test material: (**a**) An air guide tube, (**b**) pressure transmitter connector and (**c**) silicone oil.

The diffusion silicon high temperature pressure transmitter used in this device is MIK-P300G-7-BDCL type pressure transmitter produced by Hangzhou Mecon Automation Technology Co., Ltd. (Hangzhou, China) with a range of 0–4 MPa and an accuracy of 0.5 and its appearance is shown in Figure 2a; a paperless recorder of the MIK-R200T type is used. The recorder has two channels and can record the data transmitted by the pressure transmitter, thermocouple at the same time; its appearance is shown in Figure 2b As shown in Figure 2b, an electric furnace with a digital display is used; the power is 1000 W, the diameter of the furnace plate is 15 cm, and its appearance as shown in Figure 2c. It has a metal filter stainless steel gas-guide capillary as shown in Figure 2, where the length of the stainless steel gas-guide capillary is 30 cm, the inner diameter is 2 cm, the outer diameter is 4 cm. One end of which a stainless steel pressure gauge adapter is welded. The other end is welded with a cylindrical metal tray with an inner diameter of 14 mm, a metal screen with a diameter of 13 mm and a thickness of 1.7 mm, and an internal pore size of 1 μm is set at the mouth of the tray. In this thesis, a cylindrical tray is welded at one end of the gas-guided capillary tube, mainly to increase the contact area between the gas-guided tube and the inside of the cement paste test block, in order to expand the scope of collecting the internal pore pressure of the cement paste test block; the customized metal screen only allows the water vapor generated in the cement paste test block after heating to pass, and can prevent the silicone oil inside the gas-guided tube from flowing out. The water vapor entering the capillary tube through the metal screen will produce a pressure on the silicone oil filled inside the tube. Following this, the change in pore pressure inside the cement paste test block is measured by diffusion silicon high-temperature pressure transmitter and paperless recorder, and its appearance is shown in Figure 3a,b; the silicone oil used in the test is shown in Figure 3c, and its main component is polydimethylsiloxane with viscosity of 1000 cps and thermal expansion coefficient of 9.45 × 10^−4^ cm^3^/°C.

#### 2.2.3. Connection and Installation of Cement Paste Specimen and Test Device

**Figure 4:**
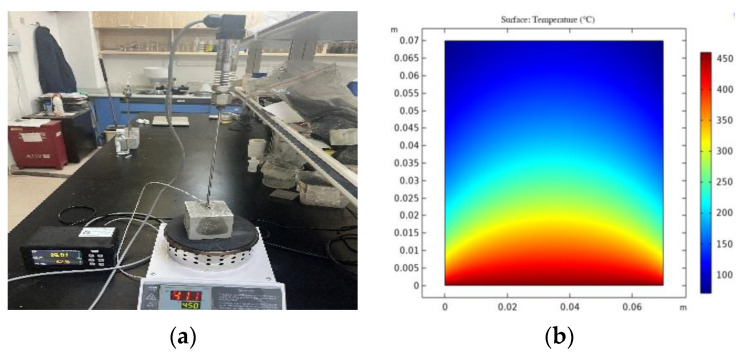
(**a**) Pore pressure test set-up; (**b**) Temperature simulation diagram.

The cement paste test block was placed in the center of the electric furnace plate, and each test apparatus was connected and assembled in turn, as shown in Figure 4a. To obtain the set temperature of the heating base plate that makes the center temperature of the upper surface of the 7-cm test block 98 degrees, the heating process of the test block was modeled. The ambient temperature in the laboratory was 20 °C. After the temperature field calculation, it was determined that setting the heating temperature of the bottom plate to 450 °C could make the surface center temperature of the test block reach 98 °C. At this time, the internal temperature distribution of the test block is shown in Figure 4b. Therefore, the heating temperature of the furnace plate was set to 450 °C for this test.

#### 2.2.4. Test Process

The whole test process lasts about 4 h. The temperature controller set the target temperature of the furnace plate, when the temperature of the furnace plate rises to 450 °C through the temperature controller’s automatic temperature control, so that the cement paste test block has a heated surface for continuous constant temperature processing, until the recorder shows the cement paste test block test point measured by the pore pressure reached the peak and began to reduce. The resistance furnace was closed to complete the entire pore-pressure-test process.

### 2.3. Mercury Compression Test of Cement Paste Specimens

#### 2.3.1. Preparation of Mercury-Pressed Specimens

The net pulp test pieces were cracked, and particles with a particle size in the range of 1–3 cm were selected and immersed in an anhydrous ethanol solution to terminate hydration. The anhydrous ethanol solution was replaced every 24 h, and the operation was repeated twice, and then the samples were dried in a vacuum drying oven for 12 h, and the temperature was set at 60 °C to finally obtain the pressed mercury specimens.

#### 2.3.2. Mercury-Pressing Experimental Apparatus

The study of pore structure characteristics was carried out by mercury-intrusion porosimetry (MIP), and the instrument used for the test was a PoreMaster 33 fully automatic mercury intrusion porosimeter manufactured by Conta Instruments of Boynton Beach, FL, USA. The low-pressure chamber was mainly used to complete the low-pressure analysis part of the test, and the pressure range was 0~345 kPa. The high-pressure chamber mainly completes the high-pressure analysis part of the test; the pressure range is from the standard atmosphere to 414 MPa. The pore-structure information and critical pore radius can be read out directly from the differential pore distribution curve.

## 3. Phase-Field Simulation of Pore Pressure Inside the Cement Paste

### 3.1. Model Building

#### 3.1.1. Assumption of Ideal Conditions

To simplify the numerical analysis study, the following ideal assumptions are made.

(a) The cement paste is considered a continuous isotropic homogeneous porous medium.
(b) It is assumed that only water vapor generates pressure in the internal pores of the cement paste, neglecting the effect of internal air (since the air content is relatively small) and considering water vapor as an ideal gas.
(c) The cement paste is assumed to be saturated and fully hydrated at room temperature (i.e., the internal hydrates are considered to no longer undergo hydration reactions).

#### 3.1.2. Pore Size Selection

**Figure 5:**
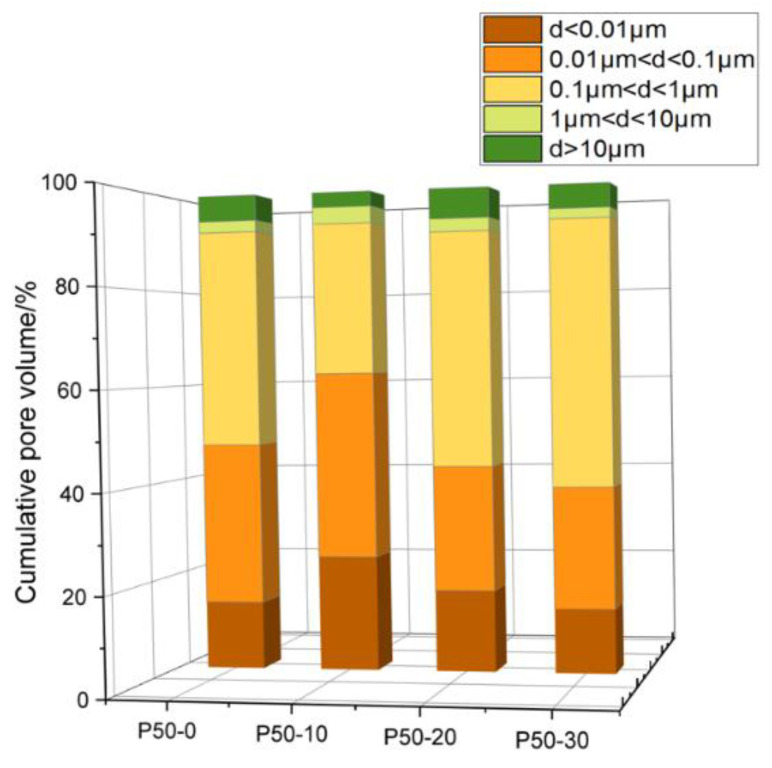
Variation in pore content of different categories of net pulp (w/c = 0.5).

According to the results of the mercury-pressure experiments, the pore size was divided into intervals, and the approximate porosity of each interval could be known by calculating the percentage share. As shown in Figure 5, the pore percentages of the specimens are mainly concentrated at d < 0.01 μm, 0.01 μm < d < 0.1 μm, and 0.1 μm < d < 1 μm. Therefore, combined with the pore size probability distribution diagram, and also for the convenience of grid division, the modeled pore diameters are taken as 0.01 μm, 0.07 μm and 0.12 μm.

#### 3.1.3. Parameter Selection

To simplify the calculation of the program, some relevant parameters common to the numerical simulation of concrete thermodynamics were selected in this thesis as shown in Table 3.

## 4. Test Results and Analysis

### 4.1. Test Results and Analysis of Cement Paste Pore Pressure under High Temperature

**Figure 6:**
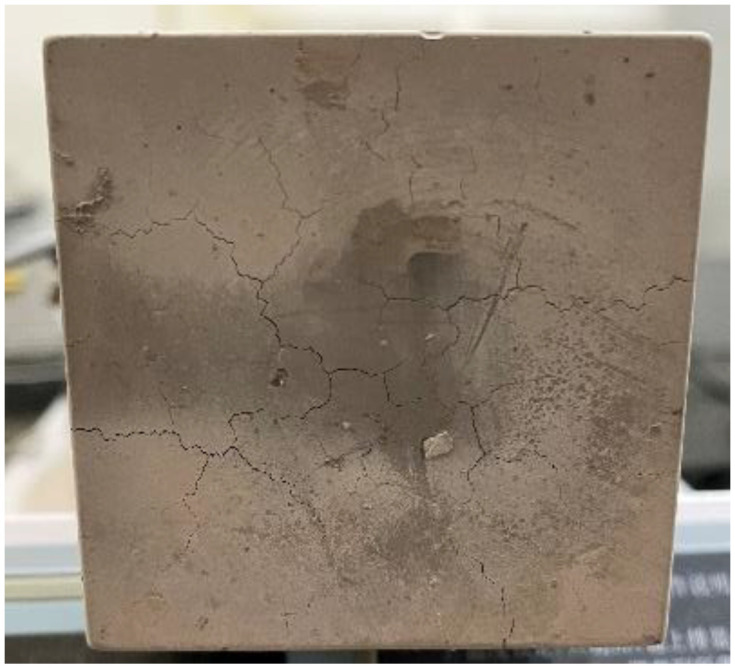
The state of the test block after the test.

During the whole test process, the pore vapor pressure test software in the cement paste automatically recorded the data, in which each group of data included the pore pressure value at the center point of the test block, and the temperature values of the heating surface and backfire surface. Through the analysis and processing of the data, the change in the law of pore pressure in the microporous structure of various test blocks under high temperatures was derived. When the specimen was heated for about 70 min, the water seepage phenomenon on the unheated surface of the specimen began to grow; after continuing to be heated for about 150 min, the water seepage on the unheated surface of the concrete began to gradually decrease; after the specimen was heated for about 200 min, the water seepage on the surface of the plate almost completely disappeared; after 4 h of high temperature heating, the whole heated surface of the specimen was scattered with fine holes, the surface layer of the central heated area was peeling off, and a small number of fine cracks appeared around the bottom surface, as shown in Figure 6.

#### 4.1.1. Temperature–Time Curve

**Figure 7:**
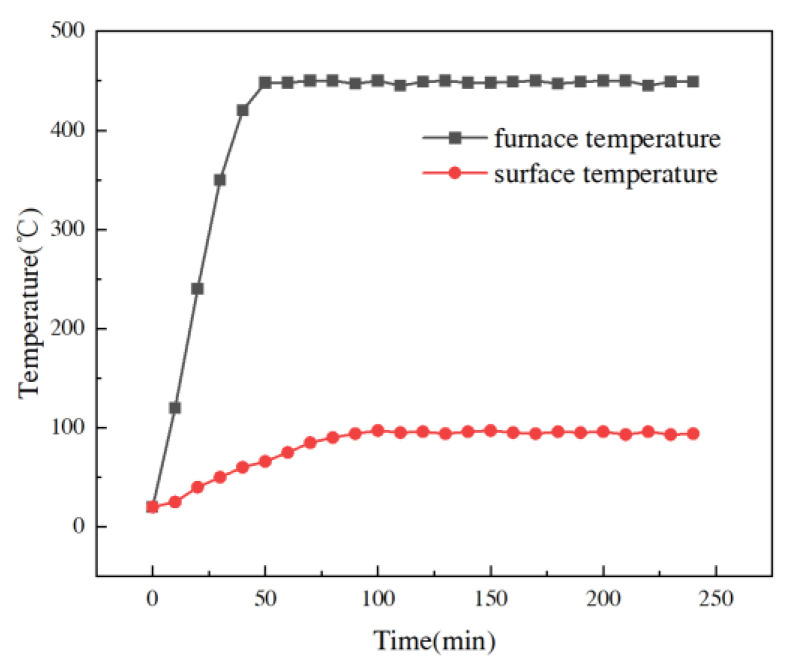
Temperature-variation curve of cement paste of test block with time.

Figure 7 shows the temperature-change curves in the heating surface of the cement paste test blocks and their relative surfaces. Since all the test blocks were heated with the temperature curves close to each other, this thesis unifies Figure 7 as the temperature–time curves of all the test blocks. The test block was placed on the furnace plate heating device, and it took about 45 min to heat from room temperature 20 °C to 450 °C during the whole test process, and the temperature of the furnace plate was maintained at 450 ± 10 °C when the temperature of the furnace plate rose to 450 °C by automatic temperature control instrument. The bottom surface of the test block and the heating bottom surface of the furnace plate formed a relatively confined space, so the temperature of the heating surface of the test block rose rapidly, and with continuous heating, the heat was gradually transferred from the heating surface to the inside of the test block, and when the heat was transferred to the backfire surface, the temperature of the backfire surface rose relatively slowly, and when the temperature rose to 98 °C, it will tend to be stable. As the backfire surface of the test block was directly exposed to the air, to a certain extent, it also increased the heat dissipation of the backfire surface of the test block, and the heat was lost with the increase in the conduction distance, which caused the temperature inside the test block to be lower the farther it was away from the heating surface.

#### 4.1.2. Pressure–Time Curve

**Figure 8:**
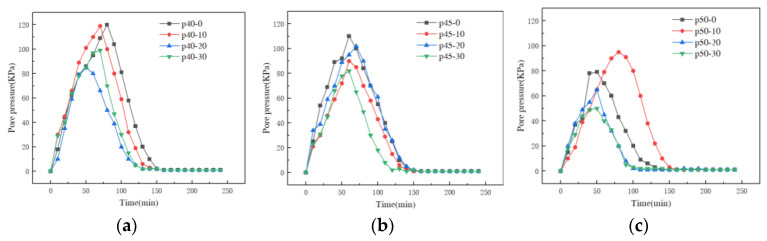
Curves of pore pressure with time for cement paste at different water-cement ratios: (**a**) w/b = 0.40, (**b**) w/b = 0.45 and (**c**) w/b = 0.50.

Figure 8 shows the pore-pressure variation curves at the center point of 12 groups of cement paste test blocks. Observing the pore pressure curves in the figure, it can be seen that after starting to heat the test blocks, the pore pressure of all the test blocks at the measurement points showed a trend of first rising and then falling, which is because with the increase in temperature, the free water and chemical water near the measurement points evaporated into water vapor, and the water vapor gradually accumulated in the closed micro-pore structure inside the concrete, resulting in the gradual increase in the pore pressure in the micro-pore structure, and a continuous increase in the pore pressure. The continuous increase in pore pressure caused micro-cracks in the micro-pore structure, resulting in the gradual penetration of numerous micro-pore structures, until these micro-pore structures penetrated the surface of the test block (i.e., cracking occurred), which provides a stable channel for water vapor to drain to the outside of the test block, and the pore pressure gradually decreases with the formation of the channel.

**Figure 9:**
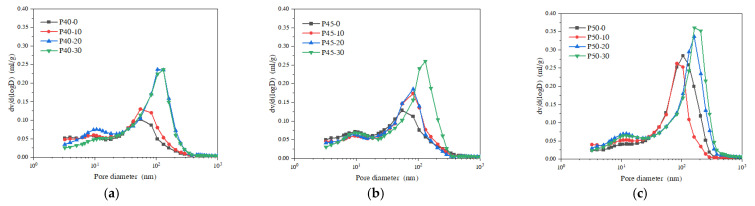
Probability distribution of pore size of cement paste with different ratios: (**a**) w/c = 0.4; (**b**) w/c = 0.45; (**c**) w/c = 0.5.

The porosity and peak pore pressure of the samples obtained by the mercury-pressure method and the time corresponding to the peak are listed in Table 4; it can be seen that the peak pore pressure is concentrated at 50~121 kPa, and the time corresponding to the peak is concentrated 50~80 min after heating, among which the peak pore pressure of P40-0 test block was the largest, at 121 kPa, and the time required to reach the peak pore pressure was the longest, at 80 min. According to the temperature–time curve, we can derive the temperature of the back side of the test block, 90 °C; the peak pore pressure of P50-30 test block was the smallest, 50 kPa, and the time corresponding to the peak was the shortest, 50 min. The temperature of the back side of the test block was 66 °C at this time. Figure 9 shows the differential pore size distribution of the piezometric mercury of cement fly ash slurry at 7 d. It can be found that (1) the pore size where the peak of the differential pore distribution of the specimen was located gradually became larger as the water–binder ratio increased. This is because the different water–binder ratios provide different growth spaces for hydration products. When the water–binder ratio decreases, it can promote the crystallization nucleation and crystal growth of hydration products, promote the degree of hydration reaction in the early stage of the net slurry, and the filling effect of hydration reaction products makes the material become more dense. (2) When the water–binder ratio ws 0.4, the total pore content increased first and then decreased with the increase in fly ash content; when the water–binder ratio was 0.45, the total pore content had a maximum value at 30% fly ash admixture, and the pore content in the d > 100 nm interval increased with the increase in fly ash admixture; when the water–binder ratio was 0.5, the total pore content decreased first and then increased, and the total pore content also increased. The total pore content had the maximum value when the fly ash mixture was 30%; at this time, the pore content in the zone of 1000 nm > d > 100 nm was the largest. This is because the physical action of fly ash and volcanic ash reaction have important effects on the pore structure of cement paste. In the early stage of hydration, the incorporation of fly ash can reduce the water requirement of cement paste and improve the hydration of cement. Therefore, the specimens with a water–binder ratio of 0.45 and 0.5 had a relatively large porosity when the fly ash admixture was 30%, and with the increase in fly ash admixture and water–binder ratio, its dilution effect and sufficient water led to a large number of connected capillaries, which increased the porosity of the specimens and then led to the change in the test value of pore pressure.

### 4.2. Effective Pore Pressure of Cement Paste Specimens with Measured Analysis

#### 4.2.1. Effective Pore Pressure of Cement-Based Materials

The solid skeleton around the pores in the cement paste is subjected to the combined action of fluid (water-air) pressure in the pores and interfacial surface tension, the combined force of which is defined as the effective pore pressure. In incompletely saturated cementitious materials, one part of the pore space is filled by water and the other part by air; all pore surfaces are covered with an adsorbed water layer. The thickness $t_{a}$ of this adsorbed water layer increases with the increase in the internal relative humidity RH Based on the experimental results, the following empirical equation was established [21]:

(1) $$t_{a}=0.385\;nm-ln\left[-ln\left(RH\right)\right]*0.189\;nm$$

Considering the effect of adsorbed water, the layer thickness $t_{a}$ can be expanded Kelvin equation to Kelvin-Cohan giving the critical radius of pores $r_{lg}$; all pores with a radius less than $r_{lg}$ are filled with pore water, while pores with a radius greater than $r_{lg}$ are filled with air. Equation (2) [21], *R* = 8.31446 J/mol·K, *T* and $\gamma^{lg}$ denotes the gas constant, the absolute temperature, temperature-dependent surface tension at the liquid-gas interface. $v_{m}$ is the molar volume of water, φ is the contact angle between the pore water and the solid skeleton; for cementitious materials the angle is usually taken as zero.

(2) $$r_{lg}=-\frac{2\gamma^{lg}v_{m}cos\varphi }{ln\left(RH\right)RT}+t_{a}$$

Depending on the pore radius, the effective pore pressure $p\left(r\right)$ can be expressed as the following equation [22]:

(3) $$p\left(r\right)=\left\{\begin{array}{ll} p_{l}-\frac{2\gamma^{sl}}{r-t_{a}}, & r\leq r_{lg}\\ p_{g}-\frac{2\gamma^{sg}}{r-t_{a}}, & r>r_{lg} \end{array}\right.$$

where $\gamma^{sl}$ and $\gamma^{sg}$ are the surface tensions at the solid–liquid and solid–gas interfaces, respectively; r denotes the pore radius; $p_{l}$ and $p_{g}$ are the pore gas pressure and pore liquid pressure, respectively; and finally, the expression of the effective pore pressure $p\left(r\right)$ is simplified as [23]:

(4) $$p\left(r\right)=\left\{\begin{array}{ll} ln\left(RH\right)\frac{RT}{v_{m}}, & r\leq r_{lg}\\ -\frac{2\gamma^{lg}}{r-t_{a}}, & r>r_{lg} \end{array}\right.$$

Solving the average effective pore pressure of the pores requires establishing the size distribution function of the pores, and the pore-size probability distribution function $\phi_{p}^{pdf}$ in the cement paste can be described by an exponential function [24], as shown in Equation (5):

(5) $$\phi_{p}^{pdf}\left(r\right)=\frac{1}{R_{p}}exp\left(-\frac{r}{R_{p}}\right)$$

The average effective pore pressure p of the resulting pores can be obtained by integrating the effective pore pressure over the respective pore size probability distribution function, as shown in Equation (6) [25]. The following key is to establish the pore size distribution function of the pore, i.e., to determine the characteristic radius $R_{p}$ of the pore in Equation (5).

(6) $$p=\frac{1}{R_{p}}\int \;p\left(r\right)\exp\left(-\frac{r}{R_{p}}\right)\mathrm{dr}$$

#### 4.2.2. Numerical Calculation Results of the Average Effective Pore Pressure

The characteristic pore sizes of the specimens maintained for seven days and obtained by mercury pressure testing are listed in Table 5. Where the characteristic pore size is the pore size corresponding to the highest point of the pore distribution, which indicates the size of the smallest of the largest interconnected pores. Due to the difficulty of measuring the relative humidity inside the pores, three RH values (0.90, 0.95, and 0.98) are taken in this paper according to the literature [26], and the effective pore pressures corresponding to the corresponding RH values are calculated separately. When the RH is 0.90, 0.95 and 0.98, respectively, the corresponding critical sizes $r_{lg}$ at room temperature are 11.02 nm, 21.95 nm and 54.45 nm, respectively, calculated by Equations (1) and (2), while the solved average effective pore pressure *p* values are listed in Table 5.

It can be found that the calculated average effective pore pressure has the same trend of change regardless of the value of RH, i.e., the average effective pore pressure decreases as the characteristic pore size gets larger. It is also found that the increment of the maximum pore pressure of the experimental test is at the level of kPa, and the average effective pore pressure of the theoretical calculation is at the level of MPa. This is because in the process of testing, the water vapor collected by the pore gas collector will condense, and the gas will rub against the capillary tube as well as the complete airtightness of the whole test equipment cannot be guaranteed, and the above situation will certainly cause damage to the pore pressure. Although there are some differences between the data obtained by the two methods, the trend of pore pressure change is the same, that is, the smaller the porosity of the sample and the smaller the characteristic pore size, the greater the pore pressure obtained from the test and the theoretical calculation of the effective pore pressure.

### 4.3. Simulation of Microscopic Crack Development by Phase-Field Method

The phase field method is a numerical method to describe the development of cracks using phase field parameters [27,28]. In the results, parameter values greater than 0.9 (red part) represent cracks, parameter values less than 0.3 (blue part) represent undamaged regions, and the intermediate transition colors represent the regions in between. Through the phase field simulation, we can simulate the process of crack eruption and development, to better understand the law of crack extension and the influencing factors.

According to the pore pressure test data, we can find that the internal pore pressure increases to a certain value first and then decreases gradually when different test blocks are warmed up. We speculate that this is due to the gradual increase in pore pressure during the warming process leading to the microscopic crack expansion and coupling, which eventually leads to the gradual equilibrium between the internal pressure and the outside world, thus leading to the decrease in pore pressure. Therefore, based on the established RVE model, we performed the phase field method simulations of crack extension for the P50-0 and P50-10 models at scale to obtain the crack extension due to the increase in internal pore pressure during warming.

The following are the results of the phase field simulations for crack initiation and development in both groups. In the simulations, we considered the effect of internal pore pressure on crack extension. The results show that with the increase in the internal pore pressure, the crack extension is gradually accelerated, while the crack extension range is also expanded. Eventually, the crack extends to the material surface, leading to damage to the test block.

(1) Crack sprouting and development in group P50-0

**Figure 10:**
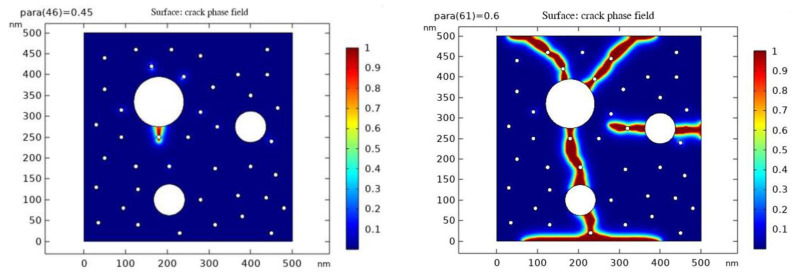
p50-0 group parameter values at crack emergence (**left**) and crack penetration (**right**).

As can be seen from Figure 10, the cracks in the P50-0 group started to sprout when the internal pore pressure increased by 45 kPa, and the cracks completely penetrated from top to bottom when the pore pressure increased by 60 kPa.

(2) P50-10 group crack sprouting and development

**Figure 11:**
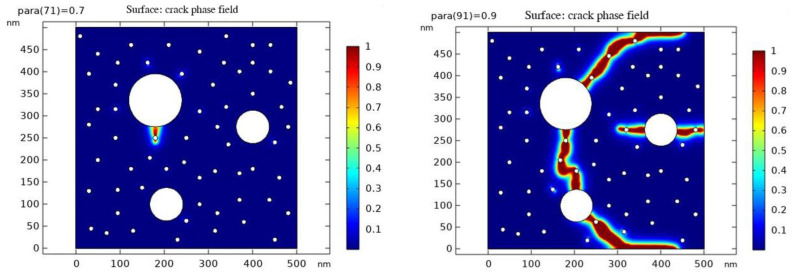
p50-10 group parameter values for crack emergence (**left**) and crack penetration (**right**).

From Figure 11, it can be seen that the cracks of the P50-10 group started to sprout when the internal pore pressure increased by 70 kPa, and the cracks were completely penetrated from top to bottom when the pore pressure increased by 90 kPa.

The crack development of the P50-0 and P50-10 groups are listed in Table 6, which shows that after the addition of 10% fly ash, there is a significant delay in the sprouting and development of cracks, which also verifies the results in the pore pressure test.

## 5. Conclusions

In this paper, with the background of a low thermal environment, firstly, the low thermal load environment, with a thermal environment from room temperature to slightly below 100 °C, was simulated, and the pore pressure value inside the pulp quantified; secondly, the porosity, characteristic pore size and different pore size distribution functions of the net pulp specimens were tested by a mercury-pressure experiment, calculating the corresponding average effective pore pressure theoretically; and finally the phase field method was used to solve the model. The temperature field and pore pressure field were solved using the phase field method, and the pore pressure variation law was analyzed by comparing the test data. The following results were obtained:

(1) As the water–binder ratio increases, the pore size where the differential pore distribution peak of the specimen is located gradually becomes larger, and the pore pressure test peak gradually decreases. This is because when the water-ash increases, the crystallization nucleation and crystal growth of hydration products are weakened, slowing down the degree of hydration reaction in the early stage of the net slurry, and the compactness of the specimen decreases, and the pore pressure required to produce cracks in the micro-pore structure becomes smaller.
(2) The physical action of the fly ash and volcanic ash reaction has important effects on the pore structure of cement paste. In the early stage of hydration, the incorporation of fly ash can reduce the water requirement of cement paste and improve the hydration of cement. When the fly ash admixture is 30%, the specimen appears relatively large with respect to porosity, and with an increase in fly ash admixture, its volcanic ash effect leads to a large number of connected capillaries, which increases the porosity of the specimen and therefore leads to a decrease in pore pressure.
(3) The larger the characteristic pore size of the net slurry, the smaller its average effective pore pressure; the theoretically calculated pore pressure value and the pore pressure value obtained from the test have the same variation trend.
(4) The results of simulating crack development by the phase field method show that when 10% fly ash is added, there is a significant delay in the sprouting and development of cracks. The results in the pore pressure test are verified: when 10% fly ash is added to the P50-0 group, its porosity decreases from 29.6% to 26.5%, the pore size where the differential pore distribution peak is located becomes smaller, the compactness of the specimen rises, and the micro-pore structure requires greater pore pressure to produce micro-cracks.

## Figures and Tables

**Table 1:** Chemical composition and physical properties of cement and fly ash.

Composition	Cement (%)	Fly Ash (%)
CaO	66.41	7.88
SiO_2_	20.99	52.50
Fe_2_O_3_	3.44	3.42
SO_3_	2.40	1.47
Al_2_O_3_	4.43	27.43
K_2_O	0.71	1.15
MgO	0.87	1.05
P_2_O_5_	0.12	0.47
Others	0.62	4.63

**Table 2:** The mix ratio of paste.

W/B	Sample	Cement (kg/m^3^)	Fly Ash (kg/m^3^)	Water (kg/m^3^)	Fly Ash Content (%)
0.4	P40-0	1368	0	547	0
	P40-10	1204	134	535	10
	P40-20	1052	263	526	20
	P40-30	900	385	514	30
0.45	P45-0	1231	0	578	0
	P45-10	1130	113	565	10
	P45-20	986	247	555	20
	P45-30	843	361	542	30
0.5	P50-0	1218	0	609	0
	P50-10	1078	120	599	10
	P50-20	941	235	588	20
	P50-30	805	345	575	30

**Table 3:** Mechanical properties and thermal parameters of the substrate.

Parameters	Value (P50-0)	Value (P50-10)
Density	1.2 g/cm^3^	1.1 g/cm^3^
Conductivity of Heat	0.65 W/m·K	0.69 W/m·K
Specific Heat Capacity	1.88 kJ/(kg·K)	2.02 kJ/(kg·K)
Modulus of Elasticity	17.4 GPa	18.5 GPa

**Table 4:** Relevant results obtained from experimental testing of the samples.

Sample	Porosity	Peak Pore Pressure (kPa)	Peak Corresponding Time (min)
P40-0	21.1088	121	80
P40-10	23.2357	119	71
P40-20	28.4297	85	50
P40-30	26.5230	99	69
P45-0	24.4409	110	61
P45-10	26.9927	90	58
P45-20	25.1176	102	70
P45-30	28.7912	82	60
P50-0	29.6131	79	50
P50-10	26.5323	95	80
P50-20	30.8705	65	54
P50-30	31.9042	50	50

**Table 5:** The parameters of the samples.

Sample	Characteristic Pore Diameter (nm)	p_RH=0.90_ (MPa)	p_RH=0.95_ (MPa)	p_RH=0.98_ (MPa)
P40-0	27.61	8.533	5.486	2.613
P40-10	27.59	8.534	5.486	2.613
P40-20	52.52	6.080	4.270	2.334
P40-30	65.46	5.336	3.843	2.196
P45-0	27.61	8.533	5.486	2.613
P45-10	41.45	6.942	4.731	2.460
P45-20	41.65	6.924	4.722	2.458
P45-30	65.55	5.332	3.840	2.19512
P50-0	52.55	6.078	4.268	2.334
P50-10	41.55	6.933	4.726	2.459
P50-20	80.45	4.696	3.455	2.052
P50-30	81.15	4.670	3.439	2.046

**Table 6:** Phase field simulation and pore pressure test data.

Sample	Crack Initiation Pore Pressure (kPa)	Crack through Hole Pressure (kPa)	Peak Pore Pressure Time (min)
P50-0	45	60	50
P50-10	70	90	80

## Data Availability

All data generated or used during the study appear in the submitted article.
